# Comparative Lipidomics Profiling of Acylglycerol from Tuna Oil Selectively Hydrolyzed by Thermomyces Lanuginosus Lipase and Candida Antarctica Lipase A

**DOI:** 10.3390/foods11223664

**Published:** 2022-11-16

**Authors:** Junyong Xuan, Zefu Wang, Qiuyu Xia, Tingyu Luo, Qingya Mao, Qinxiu Sun, Zongyuan Han, Yang Liu, Shuai Wei, Shucheng Liu

**Affiliations:** 1 College of Food Science and Technology, Guangdong Ocean University, Guangdong Provincial Key Laboratory of Aquatic Product Processing and Safety, Guangdong Province Engineering Laboratory for Marine Biological Products, Guangdong Provincial Engineering Technology Research Center of Seafood, Key Laboratory of Advanced Processing of Aquatic Product of Guangdong Higher Education Institution, Zhanjiang 524088, China; 2 Guangdong Laboratory of Southern Marine Science and Engineering (Zhanjiang), Zhanjiang 524088, China; 3 Collaborative Innovation Center for Key Technology of Marine Food Deep Processing, Dalian University of Technology, Dalian 116034, China

**Keywords:** tuna oil, hydrolyzed acylglycerol, selective lipase, lipid modification, acylglycerol composition

## Abstract

Lipase hydrolysis is an effective method to develop different functional types of lipids. In this study, tuna oil was partially hydrolyzed at 30% and 60% by *Thermomyces lanuginosus* lipase (TL 100 L) and *Candida Antarctica* lipase A (ADL), respectively, to obtain lipid-modified acylglycerols. The lipidomic profiling of the acylglycerols was investigated by UPLC-Q-TOF-MS and GC–MS to clarify the lipid modification effect of these two lipases on tuna oil. The results showed that 247 kinds of acylglycerols and 23 kinds of fatty acids were identified in the five samples. In the ADL group, the content of triacylglycerols (TAG) and diacylglycerols (DAG) increased by 4.93% and 114.38%, respectively, with an increase in the hydrolysis degree (HD), while there was a decreasing trend in the TL 100 L group. TL 100 L had a better enrichment effect on DHA, while ADL was more inclined to enrich EPA and hydrolyze saturated fatty acids. Cluster analysis showed that the lipids obtained by the hydrolysis of TL 100 L and ADL were significantly different in the cluster analysis of TAG, DAG, and monoacylglycerols (MAG). TL 100 L has strong TAG selectivity and a strong ability to hydrolyze acylglycerols, while ADL has the potential to synthesize functional lipids containing omega-3 PUFAs, especially DAG.

## 1. Introduction

Fish oil is rich in omega-3 PUFAs, such as Eicosapentaenoic acid (EPA) and Docosahexaenoic acid (DHA), which have a wide range of health benefits, including regulating lipid metabolism and cell membrane fluidity, boosting the immune system [1,2], promoting body development, enhancing brain function, as well as preventing and treating chronic diseases (cardiovascular diseases, inflammation, etc.) [3,4,5]. Recent studies have found that ω-3 PUFAs in fish oil have good therapeutic effects on the inflammatory responses induced by coronavirus [6] and that the risk of positive COVID-19 nucleic acid tests in people who consumed ω-3 PUFA supplements has slightly decreased [7].

The EPA and DHA are mixed in natural fish oil, and their total content is generally less than 30%. EPA and DHA have different effects on health. Specifically, EPA has positive effects on preventing and treating hyperlipidemia and neurodegenerative diseases due to its capability of inhibiting oxidative stress and apoptosis [8], while DHA has positive effects on neurological development, human lymphocyte function, and neutrophil function [9]. Therefore, lipases with different selectivity are applied by many in the literature to hydrolyze fish oil to obtain lipids with different ratios and contents of EPA and DHA to meet multiple user requirements.

Lipid modification by the lipase hydrolysis of fish oil is an efficient approach for enriching ω-3 PUFAs, which can not only reduce the occurrence of adverse side reactions [10] but also improve lipid composition and structure via cleaving different locations on acylglycerols by lipase [11,12], so as to obtain structural lipids that are significantly improved in terms of physicochemical properties, metabolic characteristics, and nutritional value [13]. Chen et al. compared the effects on the lipid composition after hydrolyzing cod liver oil by lipase Candida antarctica lipase B (CALB), Candida rugosa lipase (CRL), and immobilized Thermomyces lanuginosa lipase (TLIM), respectively. They found that the cod liver oil hydrolyzed by lipase CRL had the highest content of MAG and DAG, while DHA in the acylglycerols of cod liver oil, which was catalyzed by CALB and TLIM, respectively, was evenly distributed at sn-1,3 and sn-2 with no position specificity showed by lipase CRL [14]. Xia et al. found that lipase ADL had a strong preference for saturated fatty acids, especially palmitic acid, while the content of oleic acid in the acylglycerol experienced a significant increase during the hydrolysis of palm oil [15]. The variation in HD can lead to a difference in the structure of acylglycerols in fish oil. Akanbi et al. reported that EPA and DHA in acylglycerol were separated, and DHA was enriched in the acylglycerol fraction at high HD during the hydrolysis of anchovy oil by TL 100 L [16]. Akanbi et al. also found that there was a contrary trend in the content of palmitoleic acid and oleic acid in fish oil acylglycerol after hydrolysis by ADL [17]. At present, there are many reports on the enzymatic hydrolysis of fish oil, including research on process optimization and fatty acid composition analysis, but the analysis of hydrolyzed acylglycerols in these studies is not comprehensive enough. There have been few studies on the profiling of whole lipids and fatty acids in acylglycerols obtained by the hydrolysis of different lipases with different HD.

In this study, lipase TL 100 L from Thermomyces lanuginosus and lipase ADL from Candida Antarctica were used to hydrolyze tuna oil partially to obtain lipid-modified hydrolyzed acylglycerols. The differences in lipid composition and the structure of hydrolyzed acylglycerols under a different HD were analyzed by GC–MS and LC–MS. Therefore, the lipid modification effects of these two lipases on tuna oil were compared, which provides a basis for the development of lipid-modified fish oil products with different functional types.

## 2. Materials and Methods

### 2.1. Materials

Tuna oil was kindly provided by Nu-Mega Co., Ltd. (Melbourne, Victoria, Australia); selective lipase NovoCoL (ADL) and Lipozyme^®^ TL 100 L (TL 100 L) were obtained from Novozymes. Isopropanol, acetonitrile, methanol, and ammonium acetate were provided by Sigma-Aldrich Co., Ltd. and were chromatographical grade; all other reagents and chemicals were analytical grade.

### 2.2. Enzymatic Hydrolysis of Tuna Oil

The hydrolysis of tuna oil was based on Xia’s method with some modifications [18]. An amount of 5 g of tuna oil was put into a round-bottom flask, followed by lipase with an oil mass fraction of 2%. A phosphate-buffered solution with a pH of 7.0 was added at a water-oil ratio of 2:1. The flask was then filled with N_2_ and sealed with a balloon. Finally, it was placed on a magnetic temperature-controlled stirrer. Hydrolysis was carried out at 300 r/min, 40 °C.

### 2.3. Determination of Hydrolysis Degree

A total amount of 2 g of hydrolysates was put into a small beaker with 10 mL ether-ethanal (2:1), and a mixed solvent was added to dissolve the hydrolysates. Then 1–2 drops of phenolphthalein indicator were added and mixed well; this was titrated with 0.1 mol/L NaOH solution until the reaction solution turned a red hue and did not fade within 30 s (this was the titration endpoint). The hydrolysis degree (HD) of the hydrolyzed product was calculated according to Equation (1) [18].

(1) $$\mathrm{HD}\;(\%)=\frac{56.1\times C\times V}{m\times 0.35\times 187}\times 100$$

where C is the concentration of NaOH solution (mol/L); V is the amount of NaOH solution (mL); m is the mass of hydrolysates (g); 0.35 is the proportion of oil phase in the hydrolysates; 187 is the saponification value of tuna oil.

### 2.4. Extraction of Hydrolysates

The HD of the hydrolysates was regularly tested, and the hydrolysates were extracted when they were close to the low HD (30%) and the high HD (60%) by adding 2 mL of anhydrous ethanol. Firstly, the theoretical value of lye required the neutralizing of free fatty acids in the hydrolysates and was calculated by the degree of hydrolysis. After being placed in a magnetic stirrer, 0.5 mol/L KOH 30% ethanol solution was added dropwise to neutralize the free fatty acid. The titration was stopped when the pH of the hydrolyzed solution exceeded 9. The hydrolyzed products were extracted with ether and n-hexane successively. Rotary evaporation was carried out at 50 °C. The residual organic solvent was blown dry with N_2,_ and the sample was sealed and kept at 4 °C.

Samples of the tuna oil and hydrolyzed acylglycerol obtained by lipase TL 100 L and ADL at approximately 30% and 60% HDs, respectively, were labeled TL 100 L-30%, TL 100 L-60%, ADL-30%, and ADL-60%, respectively (Table 1).

### 2.5. Fourier Transform Infrared Spectroscopy (FTIR) Analysis

Based on the method of Feng et al. with slight modifications [19], infrared spectroscopic analysis of the tuna oil and its lipid-modified hydrolysates was performed using a Fourier transform infrared spectrometer (TENSOR 27, Bruker Corporation, Germany).

The analysis conditions were as follows: the resolution was 8 cm^−1^, the number of scans was 32, and the wavenumber range was 4000~400 cm^−1^. Using the KBr tablet method, the dry KBr powder and the sample were mixed and ground at a ratio of 150:1. This mixture was pressed into a transparent film by using a tablet press and then placed in an instrument for observation. 

### 2.6. Fatty Acid Composition Analysis 

The methyl esterification of the sample was performed by the method of Xia et al. [20] with slight modifications. An amount of 10 mg of the sample was dissolved in 1 mL of toluene, followed by the addition of 200 μL of 5 mg/mL internal standard (50 mg methyl dodecanoate dissolved in 10 mL of toluene), 200 μL of 1 mg/mL antioxidant (10 mg BHT dissolved in 10 mL of toluene) and 2 mL of 10% acetyl chloride-methanol solution, which was then sealed and kept at 50 °C overnight. A total of 5 mL of NaCl solution (5%, *m/v*) was added after the mixture cooled to an ambient temperature. An amount of 5 mL of n-hexane was then added to the mixture. The supernatant was taken after shaking the mixture and allowing it to stand. The supernatant was washed with an addition of 5 mL KHCO_3_ solution (2%, *m/v*). A proper amount of anhydrous sodium sulfate was added to the mixture, which was then shaken vigorously and filtered through a 0.22 μm membrane and stored at 4 °C for gas chromatographic analysis.

The fatty acid composition was measured using a TQ8040NX gas chromatography-mass spectrometer (Shimadzu Co., Ltd., Japan) equipped with an Inert Cap^®^ Pure-WAX silica capillary column (30 m × 0.25 mm, 0.25 µm). GC analytical conditions: the carrier gas was helium gas, the pressure was 54.2 kPa, the control mode was line speed, 31.5 cm/s, the total flow was 41.7 mL/min, and the column flow was 0.70 mL/min. The sample was injected in a split mode, and the split ratio was 50:1. FID: injection port temperature 250 °C, detector temperature 250 °C. Column temperature procedure: 130 °C for 5 min, then increased to 240 °C at 4 °C/min and held for 30 min.

The fatty acids in the samples were qualitatively identified by a GC–MS mass spectrometry library combined with the mixed standard of fatty acid methyl esters and quantified by the area normalization method.

### 2.7. Non-Targeted Qualitative and Quantitative Analysis of Acylglycerols

The UPLC-Q-TOF-MS analysis conditions were based on Wu’s method [21]. The pretreatment of the samples was carried out using the IPA method. Briefly, 10 mg of a sample was dissolved in 1 mL of isopropanol and vortexed for 30 s. Then, 200 μL of the mixture was taken out and dissolved in 800 μL isopropanol and vortexed for 30 s, then filtered through an organic filter membrane of 0.22 μm. After filtration, 10 μL was taken into an interpolator tube with 80 μL isopropanol and 10 μL of 10 μg/mL internal standard, which was added into the tube. After being sonicated for 10 s, the solution was injected into the instrument.

The lipid composition was determined using the UPLC 30A system (Shimadzu Corporation, Kyoto, Japan) equipped with a Phenomenex Kinetex C18 column (100 mm × 2.1 mm, 2.6 μm), coupled to a TripleTOF 6600 system (AB Sciex, Concord, ON, Canada). The injection volume was 1 μL. The flow rate was 0.40 mL/min, and the column temperature was 60 °C. Mobile phase A was a mixture of water/MeOH/ACN (1:1:1, *v/v/v*; 5 mM ammonium acetate), and mobile phase B was a mixture of IPA/ACN (5:1, *v/v*; 5 mM ammonium acetate). The gradient elution conditions were as follows: 0–0.5 min, 20% B; 0.5–1.5 min, 40% B; 1.5–3 min 60% B; 3–13 min 98% B; 13–13.1 min 20% B; 13.1–17 min 20% B.

### 2.8. Statistical Analysis

All experiments were performed in three replicates, with results expressed as the “mean ± standard deviation”. Differences with a confidence level of 95% were determined by one-way analysis of variance (ANOVA) and Tukey HSD multiple comparisons using JMP^®^ Pro 13.0.0 software (SAS, Cary, NC, USA). Cluster heatmaps for the factors controlling the five samples and the acylglycerol species were created using Origin Pro 2022 software (OriginLab Co., Northampton, MA, USA). Clustered heat maps were produced using the group average clustering method. Additionally, the distance measurement algorithm was the Pearson correlation.

## 3. Results and Analysis 

### 3.1. FTIR Analysis of Acylglycerols from Tuna Oil Hydrolyzed by Two Lipases

**Figure 1:**
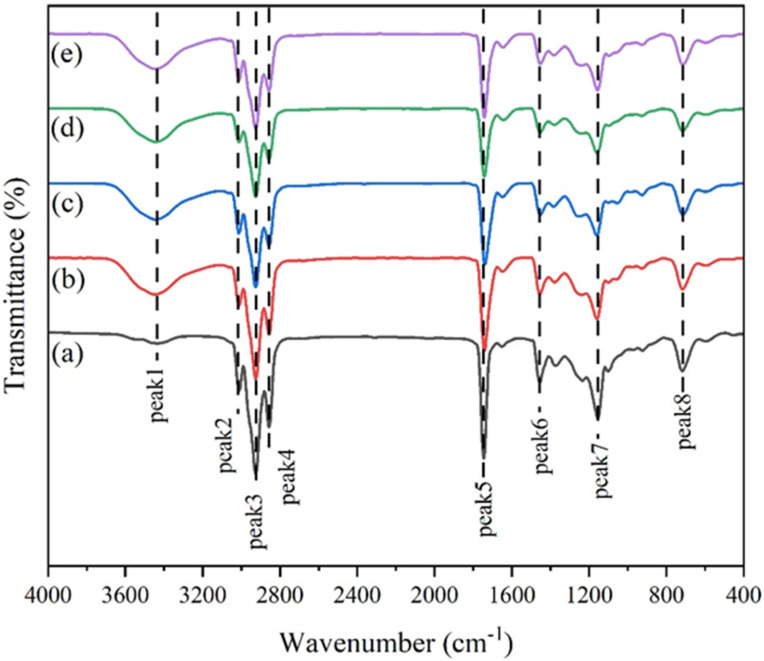
FITR spectra of tuna oil and its hydrolyzed acylglycerols: (**a**) Tuna oil; (**b**) TL 100 L−30%; (**c**) TL 100 L−60%; (**d**) ADL−30%; (**e**) ADL−60%.

The infrared spectrum of tuna oil and its hydrolyzed acylglycerols are shown in Figure 1. Additionally, eight characteristic peaks on the infrared spectrum map are listed in Table 2. Peak 1 represents the hydroxyl (-OH) stretching vibration; Peak 2 represents the cis carbon–carbon double bond (C=C) stretching vibration; Peak 3 and Peak 4 represent the asymmetric methylene (-CH_2_-) stretching vibration and symmetric methylene (-CH_2_-) stretching vibration, respectively; Peak 5 represents the axial vibration of carbonyl (C=O), which was a unique structure in the acylglycerols [22]. Peak 6 represents the in-plane deformation vibration of methylene (-CH_2_-); Peak 7 is the absorption peak of the ester bond (-COOR) due to the stretching vibration of the ether bond (-C-O-C-); Peak 8 is the long chain methylene (-CH_2_-) absorption peak [23].

The tuna oil (Figure 1a) had no obvious absorption peaks at Peak 1, while all four hydrolyzed acylglycerols (Figure 1b–e) showed broad and strong absorption peaks on Peak 1, indicating the presence of hydroxyl groups (-OH) in their group structures. This may be because tuna oil existed mainly as TAGs before hydrolysis, while DAG and MAG were generated after hydrolysis. The signal intensity of the tuna oil (Figure 1a) on Peak 2–7 was significantly greater than that of the four hydrolyzed acylglycerols (Figure 1b–e). This may be because the lipase cleaved the fatty acid esterified on the glycerol backbone during the hydrolysis of the fish oil, destroying the ether bond (-C-O-C-) so that one or two of the triacylglycerols containing a carbon–carbon double bond (C=C), methylene (-CH_2_-), and carbonyl (C=O) functional groups in the fatty acids were replaced by the hydroxyl groups. As a result, the content of the functional groups on Peak 2–7 in the four hydrolyzed acylglycerols (Figure 1b–e) decreased, and the signal intensity dropped.

### 3.2. Analysis of Fatty Acid Composition of Acylglycerols Obtained by Hydrolysis of Tuna Oil by Two Lipases

Tuna oil and its acylglycerols were detected by GC analysis to contain 23 types of fatty acids, whose compositions and contents are shown in Table 3. As can be seen from Table 3, under the catalysis of lipase TL 100 L and ADL, the carbon chain length and saturation of the two hydrolyzed acylglycerols significantly changed; the content of the saturated fatty acids, dominated by C16:0 and C18:0 in the tuna oil, was significantly reduced, and the higher the degree of hydrolysis, the lower the content. This trend is more significant after ADL hydrolysis. ADL-60% has the lowest saturated fatty acid content (reduced to 8.9% for C16:0 and 2.3% for C18:0). Yang’s study also showed that most of the free fatty acids produced during the hydrolysis of fish oil were saturated fatty acids [24]. After the hydrolysis of the fish oil, the content of polyunsaturated fatty acids, especially DHA, in the hydrolyzed acylglycerols was significantly increased, and the total content of EPA and DHA was increased to more than 1.5 times at a high degree of hydrolysis. This may be because the molecular conformation of the cis carbon–carbon double bond in EPA and DHA made the fatty acid chain bend and fold so that the methyl group at the end of the chain was very close to the ester bond, forming a steric hindrance effect, which increased the difficulty of the lipase active site to reach the ester bond of the fatty acid and its glycerol backbone [22]. As a result, the EPA and DHA were protected during the hydrolysis process. This indicates that the selective hydrolysis rate of the saturated fatty acids by these two lipases was higher than that of ω-3 PUFAs [25]. Compared with the lipase TL 100 L, ADL showed a higher selectivity and preference for the hydrolysis of the dominated saturated fatty acids, and the content of saturated fatty acid in ADL-30% and ADL-60% decreased from the initial 34.55% to 22.09% and 15.43%, respectively. The trends of the content of monounsaturated fatty acid were different in the two lipase treatment groups. In the TL 100 L group, the content of monounsaturated fatty acids decreased with an increase in HD. Conversely, ADL-30% had the highest content of monounsaturated fatty acids at 26.17% in the tuna oil and its hydrolysates. Akanbi et al. used lipase ADL to partially hydrolyze anchovy oil, and the content of monounsaturated fatty acid in the acylglycerols first increased and then decreased because ADL had a great enrichment of both monounsaturated fatty acids and polyunsaturated fatty acids at 30% HD and began to hydrolyze all the fatty acids when HD was over 40%, which is consistent with the results of this study [17]. Kazuaki et al. found that long-chain monounsaturated fatty acids combined with DHA or EPA could reduce lipid accumulation in the HepG2 cells, and C20:1 n7 when combined with DHA treatment and could significantly reduce cholesterol levels in HepG2 cells [26]. The DHA content in the TL 100 L modified acylglycerols was higher than that of the ADL-modified lipids at both a low and high HD, indicating that lipase TL 100 L had a stronger enrichment effect on DHA. Contrastingly, Lipase ADL had a stronger EPA enrichment effect. Gao et al. found that the lipase OUC-Lipase 6 derived from *Streptomyces violascens* exhibited a selectivity to enrich EPA over DHA during the hydrolysis of cod fish oil [27].

### 3.3. Lipid Composition Analysis of Acylglycerols Obtained from Hydrolysis of Tuna Oil by Two Lipases

Differences in the structure and composition of EPA and DHA in different lipid or acyl groups can affect the function of Omega-3 lipids. Polyunsaturated fatty acids in the form of acylglycerols are more easily absorbed by the body and have higher antioxidant capacity than free fatty acid and ethyl ester polyunsaturated fatty acids [28,29]. Ding et al. found that the bioavailability of DHA in the form of TAG was higher than that in other forms, which is more likely to be digested and absorbed [30]. The total content of EPA and DHA is closely related to the quality and economic value of fish oil [31]. Therefore, in order to better evaluate the modification effect of different lipases on tuna oil, the composition of acylglycerols in the tuna oil and its hydrolysates was analyzed in this study.

Through the non-targeted qualitative and quantitative analysis of lipids, it was found that 247 kinds of acylglycerols were detected in both the tuna oil and its hydrolysates, including 153 kinds of TAG, 85 kinds of DAG, 7 kinds of MAG, and 2 kinds of diacylglycerol glucuronides. TAG (16:0–16:0–22:6) and TAG (16:0–18:1–22:6) are the two most abundant acylglycerols in tuna oil, accounting for 9.15% and 8.24% of the total TAG in tuna oil, respectively. Zhang et al. [32] found that TAG (16:0–16:0–22:6) and TAG (16:0–18:1–22:6) were the representative TAG used to distinguish deep-sea fish species, which were present in much higher amounts in the tuna oil than other fish oil types. DAG (22:6–22:6) had the highest content in each hydrolysate, which is one of the reasons for the significant increase in the content of DHA after hydrolysis.

**Figure 2:**
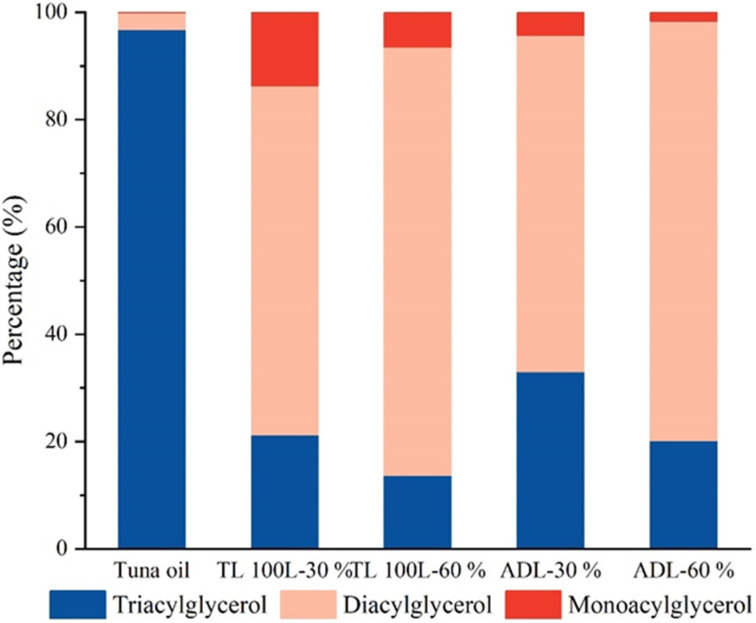
Percentage of acylglycerol composition in tuna oil and its hydrolyzed acylglycerols.

Figure 2 shows the acylglycerol composition in tuna oil and its hydrolysates hydrolyzed by TL100 and ADL, respectively (TL 100 L-30%, TL 100 L-60%, ADL-30%, and ADL-60%). It can be seen from the figure that the type of lipase and the degree of hydrolysis significantly affected the percentage of each acylglycerol in the fish oil. Before the hydrolysis of the tuna oil, the proportion of TAG in the acylglycerols reached more than 90%. After hydrolysis by the lipase, the content of TAG in the tuna oil decreased substantially, while DAG dominated. This may be because fish oil is generally hydrolyzed at the sn-1,3 position, and the catalytic triplet will preferentially hydrolyze the acyl group at the sn-1,3 position so that the diacylglycerol content increases. Morales-Medina et al. speculated that the reason the content of MAG in the fish oil hydrolysates was much less than the content of DAG was that the fatty acids on TAG were more easily cleaved by enzymatic hydrolysis than those on DAG [33]. Compared with the three other hydrolysates, TL 100 L-60%, ADL-30%, and ADL-60%, TL 100 L-30% had the highest proportion of MAG. This may be because lipase TL 100 L was a specific lipase with selectivity for sn-1,3 positions [34], which selectively hydrolyzed the fatty acids attached at sn-1 and sn-3 positions in the TAG, resulting in the formation of MAG and the enrichment of fatty acids at the sn-2 position. However, the higher the degree of hydrolysis of the tuna oil, the lower the proportion of MAG. This may be because the formation and continuous hydrolysis of MAG was carried out at the same time, but the hydrolysis rate is faster than the formation rate [35]. (Appendix A).

#### Cluster Heatmap Analysis of TAG, DAG and MAG

**Figure 3:**
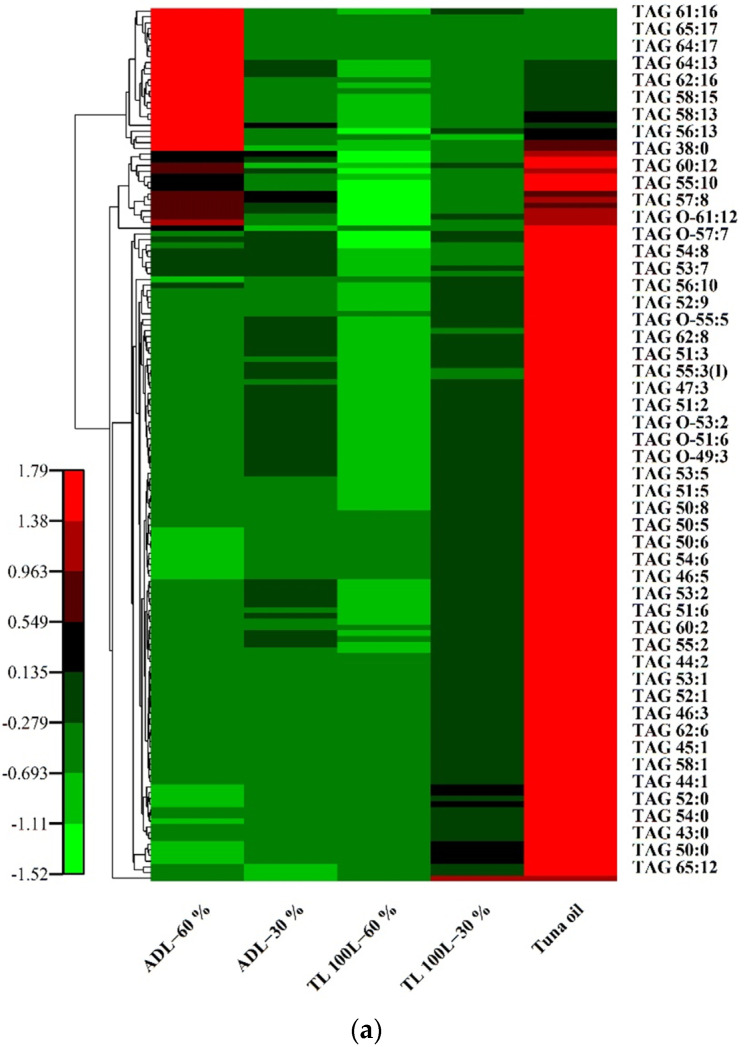

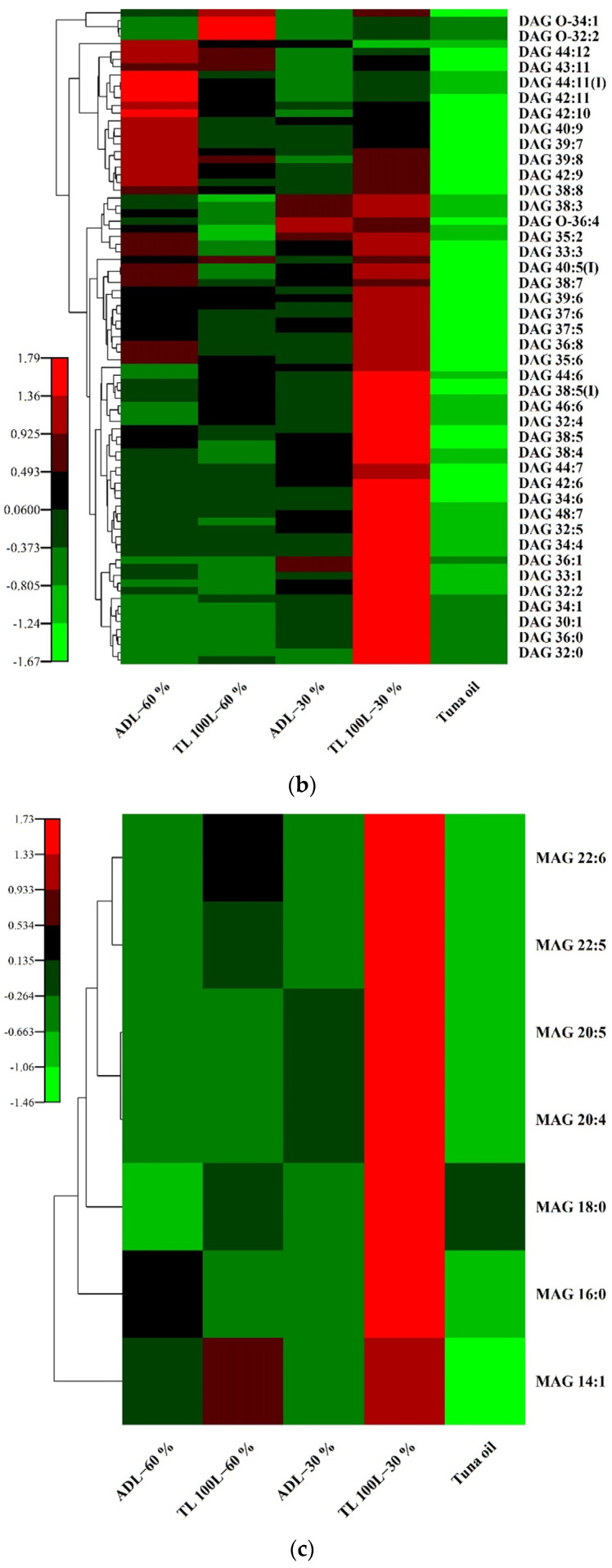
Cluster heat map of three kinds of acylglycerols. (**a**) Cluster heat map of TAG; (**b**) Cluster heat map of DAG; (**c**) Cluster heat map of MAG.

In order to better elucidate the differences in the types and composition of lipids obtained by lipase TL 100 L and ADL at low and high HD, cluster heat map analysis was used to statistically analyze the lipid composition information of the tuna oil and its hydrolyzed acylglycerols. A cluster heat map is a statistical method for classifying the combination of samples and variables. Taking the index variables as the abscissa and the sample information as the ordinate, the method of vertical comparison was adopted to visually present the global changes and clustering relationships of multi-samples and multi-variables. The cluster heat map of the three acylglycerols is shown in Figure 3.

The types of triacylglycerol can be divided into three categories (Appendix B): TAG cluster I, TAG cluster II and TAG cluster III. Specifically, TAG cluster I have 114 types of TAG, including TAG (10:0–12:0–14:0), TAG (10:0–10:0–18:1), and TAG (21:0–22:6–22:6), and these TAG were extremely high in tuna oil but low in hydrolysates. TAG cluster II has 14 kinds of TAG, including TAG (10:0–14:0–16:0), TAG (10:0–12:0–18:1), and TAG (O-17:0–22:6–22:6), and these TAG were higher in tuna oil and ADL-60% compared to the other three samples. TAG cluster III has 25 kinds of TAG, including TAG (10:0–12:0–16:0), TAG (14:1–20:5–22:6), and TAG (20:2–22:6–22:6), and these TAG were higher in ADL-60% compared to the other four samples (Figure 3a). According to the color scale of the cluster heat map, for lipase TL 100 L, the content of TAG cluster I and TAG cluster II decreased sharply at a low HD, showing the hydrolysis preference for these two types of TAG; conversely, at a high HD, it continued to hydrolyze almost every triacylglycerol. For lipase ADL, its ability to hydrolyze TAG at a low HD was not significantly different from that of TL 100 L, but it significantly increased the content of 48 kinds of TAG, including TAG cluster III. A total of 46 out of these 48 kinds of TAG contained EPA or DHA at a high HD. This may be due to the esterification or transesterification of free fatty acids in the hydrolysis system, especially free forms of EPA and DHA, with TAG at a high HD generating TAG, including TAG (14:1–20:5–22:6), TAG (20:2–22:6–22:6), and TAG (18:4–18:4–20:5). The fatty acid distribution of the TAG cluster III might have been rearranged and improved due to the regiospecificity of the lipase ADL [36]. Wang et al. used immobilized lipase MAS1 derived from the marine *Streptomyces* sp. strain W007 to catalyze the esterification of glycerol with ω-3 PUFAs and successfully prepared TAG, which was high purity (92.26%) and rich in ω-3 PUFAs. The fatty acid composition of the products was similar to that of ω-3 PUFAs, which were used as reaction substrates [37]. The location distribution of EPA and DHA on TAG also plays a key role in their digestion and absorption in the human body [38]. Combined with a lower saturated fatty acid content (15.43%) (Table 3) and its lipid composition, ADL-60% has the ability to be used as a structured lipid for omega-3 food fortification. Therefore, compared to TL 100 L, lipase ADL has a higher potential for the lipid modification of fish oil to produce lipids with specific functionalities.

The diacylglycerol species can be divided into three categories (Appendix B): DAG cluster I, DAG cluster II, and DAG cluster III. Specifically, DAG cluster I have 62 types of DAG, including DAG (14:0–16:0), DAG (16:0–16:0), and DAG (16:0–18:0), and these DAG were higher in TL 100 L-30% compared to the other four samples. DAG cluster II has 21 kinds of DAG, including DAG (16:2–22:6), DAG (18:4–20:5), and DAG (20:3–22:6), and these DAG were higher in ADL-60% compared to the other four samples. DAG cluster III has four kinds of DAG, including DAG (O-17:0–15:2), DAG (O-19:1–15:1), and DAG (O-19:0–15:1), and the content of these DAG in TL 100 L-60% was higher compared to the other four samples (Figure 3b). According to the color scale of the cluster heat map, compared with the other four samples, TL 100 L-30% contained a higher content of DAG cluster I and a lower content of DAG cluster II and cluster III, and TL 100 L-60% had a highest content of DAG cluster III. This shows that the degree of hydrolysis had a significant effect on the lipid composition. The effects of lipase ADL and TL 100 L were different. The content of the three categories of DAG in ADL-30% was generally not high, but the content of DAG cluster II increased in ADL-60%. There are 21 DAGs in DAG cluster II, 20 of which contain EPA or DHA, indicating that ADL has the potential to prepare omega-3-rich DAGs.

The seven kinds of MAG include MAG 14:1, MAG 16:0, MAG 18:0, MAG 20:4, MAG 20:5, MAG 22:5, and MAG 22:6, which had the highest content in TL 100 L-30% (Appendix B). Among the seven kinds of MAG, the content of MAG 22:6 accounted for 78.84% of the total content of MAG, which indicated that TL 100 L had better enrichment for containing DHA at a low HD. ADL had less effect on the monoacylglycerols content with a change in HD (30% and 60%), suggesting that TL 100 L had a stronger ability to hydrolyze than ADL. This result indicated that moderate hydrolysis contributed to the enrichment of acylglycerols containing DHA. DHA in fish oil was usually distributed at the sn-2 position [39], which might also indicate that TL 100 L was more inclined to hydrolyze the sn-1 and sn-3 positions. This result was consistent with the analysis of the percentage of acylglycerol composition (Figure 2).

## 4. Discussion

As can be seen from Figure 3a, in TAG cluster III, except for TAG 38:0 and TAG 56:12, the content of the other 23 TAGs did not change significantly under the hydrolysis of lipase. The reason for this may be that these TAGs are linked to at least one long-chain polyunsaturated fatty acid dominated by EPA or DHA, and the steric hindrance formed by these fatty acids is more resistant to lipase hydrolysis, while saturated fatty acids and monounsaturated fatty acids have linear and nearly linear structures, respectively, which do not form any obstacle to lipase and are easy to be hydrolyzed [39]. The types of lipases and HD have significant differences in selectivity to TAG. As can be seen from the changes in the color scale, the content of 17 kinds of saturated TAG decreased significantly at a low HD, and the effect of lipase ADL in reducing their content was stronger than that of TL 100 L, which is consistent with the results in Table 3. In 100 L-30%, the content of TAG clusters I and II decreased greatly, and the content of DAG cluster I increased greatly, and it had the highest content of MAG. It is speculated that lipase TL 100 L mainly acts on TAG clusters I and II, and the hydrolysis products are mainly DAG cluster I and all of the MAG. The distribution of DAG in ADL-30% was relatively uniform, which may be due to the fact that lipase ADL was between the selectivity of the sn-2 position and the non-selectivity position, and the production of DAG abounded in randomness [40]. In addition, the lipase preferentially hydrolyzed saturated fatty acids and began to hydrolyze all the fatty acids indiscriminately when a certain degree of hydrolysis was reached, showing fatty acid selectivity, not regioselectivity [17]. The selective hydrolysis of fish oil may be accompanied by esterification and transesterification. In the process of increasing the degree of hydrolysis from 30% to 60%, the content of 48 kinds of TAG increased in the hydrolysis reaction catalyzed by lipase ADL, while the content of only seven kinds of TAG increased in the hydrolysis reaction catalyzed by TL 100 L, and this phenomenon was concentrated on TAG with carbon numbers of 55 to 66 (also unsaturated TAG). This may be due to the presence of esterification or transesterification. TAG with higher carbon numbers was more prone to these reactions, and ADL was more capable of causing these possible reactions than TL 100 L. Therefore, the mechanism of lipase-catalyzed esterification or transesterification may be determined by a combination of the nature of the lipase itself, the type of substrate, the degree of hydrolysis of TAG, the regional distribution of fatty acids in the glycerol backbone, and the unsaturation number or chain length of fatty acids.

Hydrolysis with TL 100 L and ADL improves the tuna oil’s polyunsaturated fatty acid profile due to the modification of lipid (DHA and EPA) composition and distribution, which provides the potential to produce healthier lipids with modified properties; these new lipids may be a useful new ingredient for nutritional supplements in human or animal feeding. However, more research is needed to illustrate the functional characteristics, especially the physicochemical properties, metabolic characteristics, and nutritional value of modified tuna oil.

## 5. Conclusions

In this study, the lipid modification effect of two lipases on tuna oil at a low and high HD was evaluated by UPLC-Q-TOF-MS and GC–MS. Lipase TL 100 L showed a better enrichment of DHA, while ADL was more inclined to hydrolyze saturated fatty acids and enrich EPA. The hydrolysis reaction catalyzed by lipase ADL might cause the esterification reaction of glycerides or the acid hydrolysis reaction in transesterification. In addition, the selectivity of the two lipases in the tuna oil fatty acids varied significantly at different HDs. TL 100 L had strong specificity for sn-1,3 fatty acid and had a strong ability to continuously hydrolyze acylglycerols, while ADL exhibited the potential to produce omega-3 functional lipids due to its selective hydrolysis of saturated fatty acids and higher yield level of omega-3-containing DAGs. The specific sites of action of the two lipases and the fatty acid composition at the sn-2 position can then be further explored for in-depth analysis. This study provides a scientific basis for designing specific acylglycerol compositions and fatty acid ratios through lipid modification.

## Figures and Tables

**Table 1:** Description of the four samples.

Resource Before Hydrolysis	Sample After Hydrolysis	Lipase for Hydrolysis	HD
Tuna oil	TL 100 L-30%	TL 100 L	30%
	TL 100 L-60%	TL 100 L	60%
	ADL-30%	ADL	30%
	ADL-60%	ADL	60%

**Table 2:** Eight characteristic peaks of tuna oil and its hydrolyzed acylglycerols.

No.	Wavelength	Infrared Spectrum
Peak 1	3600–3400 cm^−1^	-OH
Peak 2	3100–3000 cm^−1^	C=C
Peak 3	3000–2900 cm^−1^	-CH_2_-
Peak 4	2900–2800 cm^−1^	-CH_2_-
Peak 5	1800–1700 cm^−1^	C=O
Peak 6	1500–1400 cm^−1^	-CH_2_-
Peak 7	1200–1100 cm^−1^	-C-O-C-
Peak 8	near 725 cm^−1^	-CH_2_-

**Table 3:** Fatty acid composition of tuna oil and its hydrolyzed acylglycerols.

Fatty Acids	Normalized Percentage/%				
	Tuna Oil	TL 100 L-30%	TL 100 L-60%	ADL-30%	ADL-60%
C14:0	4.34 ± 0.21 ^a^	3.57 ± 0.11 ^a^	2.70 ± 0.18 ^b^	2.48 ± 0.14 ^bc^	1.75 ± 0.42 ^c^
C14:1	0.44 ± 0.33 ^a^	0.23 ± 0.05 ^a^	0.27 ± 0.22 ^a^	0.15 ± 0.07 ^a^	0.29 ± 0.17 ^a^
C15:0	1.17 ± 0.07 ^a^	1.01 ± 0.13 ^a^	0.89 ± 0.16 ^ab^	0.78 ± 0.10 ^ab^	0.53 ± 0.12 ^b^
C16:0	20.88 ± 0.53 ^a^	17.19 ± 0.09 ^b^	14.17 ± 0.53 ^c^	13.05 ± 0.35 ^c^	8.90 ± 1.39 ^d^
C16:1 n7	5.41 ± 0.12 ^a^	4.41 ± 0.22 ^ab^	3.65 ± 0.39 ^b^	5.48 ± 0.36 ^a^	4.10 ± 0.45 ^b^
C17:0	1.47 ± 0.11 ^a^	1.00 ± 0.11 ^b^	0.98 ± 0.07 ^b^	1.01 ± 0.11 ^b^	0.84 ± 0.11 ^b^
C17:1	0.64 ± 0.12 ^a^	0.60 ± 0.08 ^a^	0.59 ± 0.11 ^a^	0.76 ± 0.10 ^a^	0.63 ± 0.20 ^a^
C18:0	5.82 ± 0.01 ^a^	4.47 ± 0.24 ^b^	3.36 ± 0.21 ^c^	3.71 ± 0.08 ^bc^	2.29 ± 0.47 ^d^
C18:1 n9	15.39 ± 0.26 ^ab^	12.87 ± 0.59 ^bc^	10.92 ± 1.21 ^c^	17.73 ± 0.65 ^a^	14.93 ± 1.17 ^ab^
C18:2 n6	1.51 ± 0.03 ^a^	1.21 ± 0.01 ^b^	1.15 ± 0.05 ^b^	1.69 ± 0.03 ^a^	1.56 ± 0.16 ^a^
C18:3 n6	0.28 ± 0.13 ^a^	0.22 ± 0.14 ^a^	0.21 ± 0.05 ^a^	0.20 ± 0.09 ^a^	0.28 ± 0.10 ^a^
C18:3 n3	0.45 ± 0.11 ^a^	0.43 ± 0.11 ^a^	0.33 ± 0.08 ^a^	0.56 ± 0.04 ^a^	0.45 ± 0.13 ^a^
C20:0	0.29 ± 0.14 ^a^	0.25 ± 0.06 ^a^	0.45 ± 0.52 ^a^	0.49 ± 0.35 ^a^	0.54 ± 0.43 ^a^
C20:1	1.09 ± 0.22 ^ab^	0.97 ± 0.12 ^ab^	0.72 ± 0.16 ^b^	1.25 ± 0.11 ^a^	0.86 ± 0.09 ^ab^
C20:2 n6	0.35 ± 0.11 ^a^	0.26 ± 0.07 ^a^	0.27 ± 0.11 ^a^	0.22 ± 0.06 ^a^	0.23 ± 0.06 ^a^
C20:4 n6	2.72 ± 0.49 ^ab^	2.56 ± 0.11 ^b^	2.15 ± 0.17 ^b^	2.88 ± 0.02 ^ab^	3.52 ± 0.19 ^a^
C20:3 n3	0.36 ± 0.17 ^a^	0.20 ± 0.08 ^a^	0.29 ± 0.27 ^a^	0.20 ± 0.03 ^a^	0.19 ± 0.08 ^a^
C20:5 n3	7.14 ± 0.04 ^cd^	7.83 ± 0.34 ^bc^	6.36 ± 0.79 ^d^	8.82 ± 0.15 ^b^	10.61 ± 0.41 ^a^
C22:0	0.31 ± 0.03 ^a^	0.21 ± 0.08 ^a^	0.36 ± 0.15 ^a^	0.32 ± 0.14 ^a^	0.30 ± 0.17 ^a^
C24:0	0.27 ± 0.07 ^a^	0.18 ± 0.01 ^a^	0.31 ± 0.08 ^a^	0.24 ± 0.01 ^a^	0.28 ± 0.06 ^a^
C22:5 n3	2.10 ± 0.10 ^b^	2.54 ± 0.14 ^b^	3.25 ± 0.02 ^a^	2.40 ± 0.09 ^b^	3.11 ± 0.25 ^a^
C22:6 n3	27.14 ± 0.11 ^c^	37.21 ± 0.48 ^b^	45.88 ± 2.40 ^a^	34.75 ± 0.15 ^b^	42.89 ± 1.95 ^a^
C24:1	0.43 ± 0.13 ^a^	0.59 ± 0.07 ^a^	0.75 ± 0.52 ^a^	0.80 ± 0.75 ^a^	0.93 ± 0.81 ^a^
SFA	34.55 ± 1.16 ^a^	27.87 ± 0.84 ^b^	23.20 ± 1.90 ^c^	22.09 ± 1.28 ^c^	15.43 ± 3.17 ^d^
MUFA	23.39 ± 1.19 ^ab^	19.67 ± 1.13 ^cd^	16.90 ± 2.61 ^d^	26.17 ± 2.04 ^a^	21.73 ± 2.89 ^bc^
PUFA	42.06 ± 1.30 ^c^	52.46 ± 1.47 ^b^	59.90 ± 3.95 ^a^	51.74 ± 0.66 ^b^	62.84 ± 3.32 ^a^
EPA + DHA	34.28 ± 0.15 ^c^	45.04 ± 0.82 ^b^	52.24 ± 3.19 ^a^	43.58 ± 0.30 ^b^	53.50 ± 2.37 ^a^

Note: SFA is saturated fatty acid; MUFA is monounsaturated fatty acid; PUFA is polyunsaturated fatty acid; Mean values in the same row with different superscripts are significantly different (*p* < 0.05).

**Figure A1:**
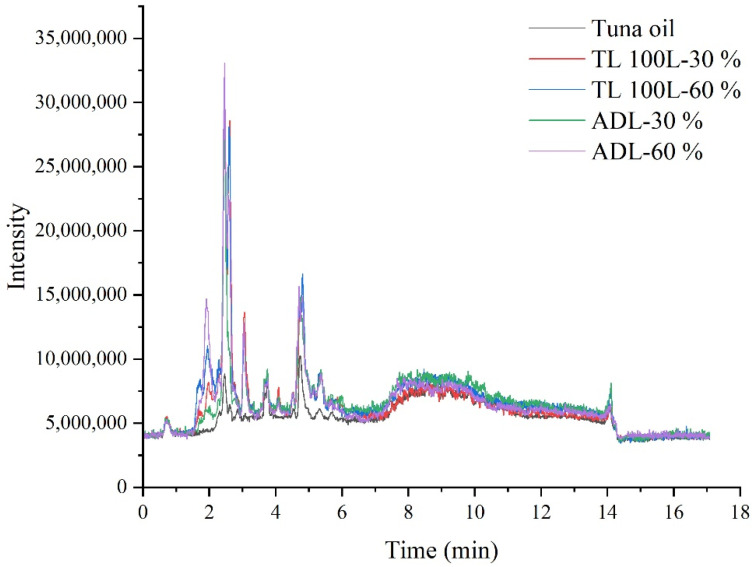
UPLC-Q-TOF-MS negative ion mode of five samples.

**Figure A2:**
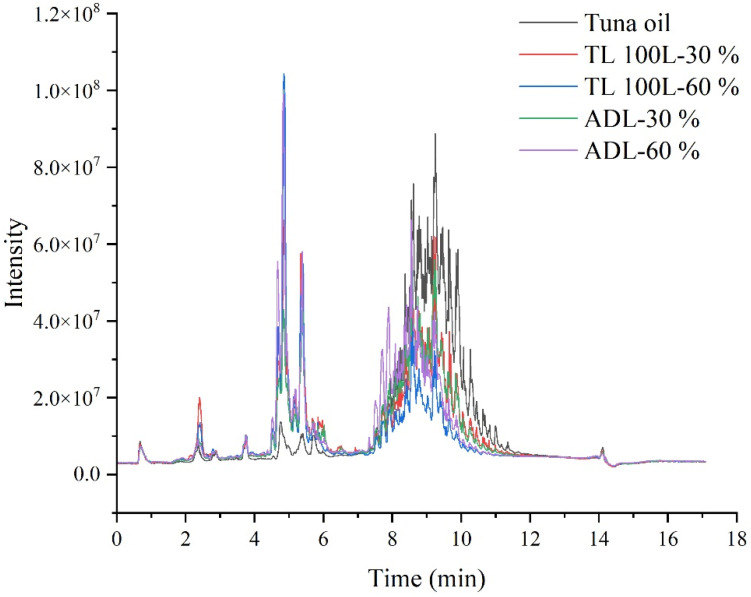
UPLC-Q-TOF-MS positive ion mode of five samples.

## Data Availability

The datasets generated for this study are available on request to the corresponding author.

## Appendix B

**Table A1:** Composition and content of acylglycerols in five samples.

NO.	Acylglycerols	Tuna Oil	TL 100 L-30%	TL 100 L-60%	ADL-30%	ADL-60%
		Content (μg/g)				
1	DAG 30:0; DAG 14:0–16:0	47.32	475.13	74.70	45.69	20.12
2	DAG 30:1; DAG 14:0–16:1	38.63	339.82	55.14	101.13	66.83
3	DAG 32:0; DAG 16:0–16:0	125.00	1326.75	145.40	106.33	63.97
4	DAG 32:1; DAG 16:0–16:1	211.87	2111.23	314.95	705.38	318.29
5	DAG 32:2; DAG 16:1–16:1	69.96	722.03	136.78	372.39	220.36
6	DAG 32:3; DAG 14:0–18:3	23.75	351.98	116.93	139.56	117.21
7	DAG 32:4; DAG 14:0–18:4	26.20	532.66	227.40	146.72	130.25
8	DAG 32:5; DAG 16:1–16:4	6.85	140.48	53.47	76.34	52.84
9	DAG 33:1; DAG 16:0–17:1	90.58	864.33	142.32	352.42	251.53
10	DAG 33:3; DAG 16:1–17:2	14.74	302.33	103.35	214.25	233.33
11	DAG 34:0; DAG 16:0–18:0	73.61	522.74	78.53	113.21	57.39
12	DAG 34:1; DAG 16:0–18:1	403.19	4365.69	469.57	1487.53	684.84
13	DAG 34:3; DAG 16:0–18:3	77.77	1114.17	284.68	636.82	479.36
14	DAG 34:4; DAG 16:0–18:4	100.07	2537.22	838.75	858.07	693.11
15	DAG 34:5; DAG 14:0–20:5	113.58	3240.54	1029.56	1081.24	1140.68
16	DAG 34:6; DAG 12:0–22:6	0.00	473.72	210.62	195.98	191.63
17	DAG 35:0; DAG 16:0–19:0	0.00	85.27	14.26	20.37	0.00
18	DAG 35:1; DAG 17:0–18:1	39.99	448.56	66.25	296.99	137.22
19	DAG 35:2; DAG 17:1–18:1	67.48	809.33	158.66	691.64	669.88
20	DAG 35:3; DAG 18:1–17:2	26.63	853.13	271.65	662.56	708.50
21	DAG 35:4; DAG 15:0–20:4	23.63	561.66	204.49	316.99	329.38
22	DAG 35:5; DAG 15:0–20:5	40.19	1267.39	512.74	545.52	680.90
23	DAG 35:6; DAG 13:0–22:6	0.00	488.01	322.26	229.01	375.54
24	DAG 36:0; DAG 18:0–18:0	24.91	113.92	21.70	32.83	17.04
25	DAG 36:1; DAG 18:0–18:1	115.43	1122.42	126.34	692.08	255.61
26	DAG 36:2; DAG 18:1–18:1	219.95	2370.38	342.97	2470.80	1745.08
27	DAG 36:3; DAG 18:1–18:2	120.07	1445.63	332.78	1105.70	831.67
28	DAG 36:4; DAG 16:0–20:4	259.56	4738.41	1400.83	2228.70	1817.08
29	DAG 36:5; DAG 16:0–20:5	651.03	15,470.85	4713.80	6322.88	5960.53
30	DAG 36:6; DAG 14:0–22:6	530.49	23,909.82	13595.62	7618.44	8534.96
31	DAG 36:7; DAG 14:1–22:6	26.27	1036.15	563.22	621.78	859.90
32	DAG 36:8; DAG 16:3–20:5	5.33	353.27	185.74	204.54	267.53
33	DAG 37:5; DAG 17:1–20:4	77.19	2216.63	1002.70	1269.67	1467.85
34	DAG 37:6; DAG 15:0–22:6	176.10	6138.33	3292.58	3204.83	3917.36
35	DAG 38:10; DAG 16:4–22:6	19.73	781.55	727.54	437.00	681.68
36	DAG 38:2; DAG 18:1–20:1	51.11	479.43	82.20	433.78	221.24
37	DAG 38:3; DAG 18:1–20:2	34.50	406.95	112.38	292.39	218.78
38	DAG 38:4; DAG 18:0–20:4	168.46	1908.06	629.20	1110.65	960.27
39	DAG 38:5; DAG 16:0–22:5	471.89	10,335.06	3948.31	5561.04	5516.33
40	DAG 38:5; DAG 18:1–20:4	143.43	5158.54	3224.51	2158.30	2053.61
41	DAG 38:6; DAG 16:0–22:6	2389.80	102,309.97	66,519.14	39,001.33	39,865.78
42	DAG 38:7; DAG 16:1–22:6	620.30	25,416.58	17,033.00	18,258.36	23,891.90
43	DAG 38:8; DAG 16:2–22:6	67.72	3034.83	2213.23	1604.53	3121.21
44	DAG 38:9; DAG 18:4–20:5	32.09	1950.56	1374.73	1122.22	2153.30
45	DAG 39:6; DAG 17:0–22:6	196.61	4495.93	3149.77	2976.09	3028.51
46	DAG 39:7; DAG 17:1–22:6	240.34	6981.90	5434.90	5825.93	11,082.13
47	DAG 39:8; DAG 17:2–22:6	48.73	4188.93	4088.21	2013.50	4939.27
48	DAG 40:1; DAG 16:0–24:1	38.21	213.94	28.78	62.27	26.14
49	DAG 40:10; DAG 18:4–22:6	181.91	12,037.29	9686.87	7011.13	15,050.05
50	DAG 40:2; DAG 18:1–22:1	21.41	263.89	35.75	130.91	64.95
51	DAG 40:5; DAG 18:0–22:5	90.23	1582.24	737.01	1025.35	1470.10
52	DAG 40:5; DAG 18:0–22:5	90.23	1582.24	737.01	1025.35	1470.10
53	DAG 40:6; DAG 18:0–22:6	861.11	18,743.87	10323.01	11,102.21	10,724.67
54	DAG 40:7; DAG 18:1–22:6	1410.22	61,949.47	46509.63	54,822.89	77,305.10
55	DAG 40:8; DAG 18:2–22:6	206.87	7705.16	5693.55	5729.69	10,315.99
56	DAG 40:9; DAG 18:3–22:6	150.55	6393.74	4850.48	4169.88	9355.75
57	DAG 42:10; DAG 20:4–22:6	348.28	20,863.81	18,764.26	12,384.39	34,427.13
58	DAG 42:11; DAG 20:5–22:6	747.84	45,571.15	50,662.00	27,924.17	96,552.23
59	DAG 42:6; DAG 20:0–22:6	33.21	1239.30	587.09	649.15	541.44
60	DAG 42:7; DAG 20:1–22:6	145.09	4610.14	2406.69	2828.37	3043.95
61	DAG 42:8; DAG 20:2–22:6	51.05	1950.87	1294.44	1026.49	1132.87
62	DAG 42:9; DAG 20:3–22:6	84.50	2958.85	2249.54	1859.50	3529.76
63	DAG 43:10; DAG 21:4–22:6	0.00	255.09	282.47	177.19	412.86
64	DAG 43:11; DAG 21:5–22:6	41.22	1889.05	2404.86	935.20	2465.08
65	DAG 43:6; DAG 21:0–22:6	9.26	265.82	124.87	113.56	62.18
66	DAG 43:7; DAG 21:1–22:6	0.00	146.73	62.36	67.33	101.03
67	DAG 44:10; DAG 22:4–22:6	59.25	2464.97	2312.16	1440.98	3052.70
68	DAG 44:11; DAG 22:5–22:6	197.72	13,152.21	18,262.20	6914.77	32,316.54
69	DAG 44:11; DAG 22:5–22:6	197.72	13,152.24	18,262.24	6914.78	32,316.60
70	DAG 44:12; DAG 22:6–22:6	1260.03	139,821.33	231,409.93	68,627.77	250,527.74
71	DAG 44:6; DAG 22:0–22:6	14.11	526.51	318.57	205.98	148.49
72	DAG 44:7; DAG 22:1–22:6	37.84	1184.01	608.59	719.51	600.11
73	DAG 44:8; DAG 22:2–22:6	32.33	303.32	190.76	172.83	211.81
74	DAG 46:12; DAG 22:6–24:6	23.17	563.99	757.00	367.34	859.42
75	DAG 46:6; DAG 24:0–22:6	7.04	430.98	191.84	189.49	88.21
76	DAG 46:7; DAG 24:1–22:6	43.16	1383.75	644.44	644.77	493.10
77	DAG 46:8; DAG 24:2–22:6	21.92	162.61	115.59	115.98	79.02
78	DAG 48:12; DAG 22:6–26:6	84.86	172.97	165.86	117.27	300.08
79	DAG 48:7; DAG 26:1–22:6	17.72	209.00	78.23	106.95	85.33
80	DAG O-30:2; DAG O-17:1–13:1	6.87	10.63	31.83	30.95	42.23
81	DAG O-32:2; DAG O-17:0–15:2	12.79	51.55	187.35	21.23	23.01
82	DAG O-34:1; DAG O-19:0–15:1	3.81	20.56	93.23	4.52	4.32
83	DAG O-34:2; DAG O-19:1–15:1	29.88	88.77	335.12	34.06	46.17
84	DAG O-36:3; DAG O-19:1–17:2	206.83	356.41	376.77	262.99	271.34
85	DAG O-36:4; DAG O-19:2–17:2	94.21	283.04	146.66	312.97	215.63
86	DAGGA 34:1; DAGGA 16:0–18:1	49.68	95.98	102.69	99.96	116.57
87	DAGGA 36:2; DAGGA 18:1–18:1	258.76	370.96	287.25	371.22	351.11
88	TAG 36:0; TAG 10:0–12:0–14:0	22.62	22.02	17.63	16.13	17.46
89	TAG 38:0; TAG 10:0–12:0–16:0	19.65	17.12	16.53	16.37	21.88
90	TAG 38:1; TAG 10:0–10:0–18:1	21.64	15.57	13.24	11.23	13.67
91	TAG 40:0; TAG 10:0–14:0–16:0	30.54	21.04	21.53	19.45	26.04
92	TAG 40:1; TAG 10:0–12:0–18:1	17.62	14.50	13.02	14.66	17.53
93	TAG 42:0; TAG 12:0–14:0–16:0	108.35	39.03	25.54	25.76	24.23
94	TAG 42:1; TAG 10:0–14:0–18:1	42.97	20.95	16.59	17.18	20.13
95	TAG 43:0; TAG 14:0–14:0–15:0	77.20	24.44	11.33	11.00	9.51
96	TAG 44:0; TAG 14:0–14:0–16:0	598.74	184.92	58.24	44.77	23.91
97	TAG 44:1; TAG 14:0–14:0–16:1	417.01	99.31	41.51	64.52	36.68
98	TAG 44:2; TAG 14:0–14:1–16:1	71.69	21.92	12.89	21.33	14.04
99	TAG 45:0; TAG 14:0–15:0–16:0	319.72	117.00	37.38	39.57	18.54
100	TAG 45:1; TAG 14:0–15:0–16:1	325.19	93.39	29.93	62.54	28.15
101	TAG 45:2; TAG 15:0–14:1–16:1	83.58	27.40	11.36	24.13	16.06
102	TAG 46:0; TAG 14:0–16:0–16:0	1772.31	737.54	177.23	140.50	46.62
103	TAG 46:1; TAG 14:0–16:0–16:1	3107.62	722.24	175.80	372.83	108.81
104	TAG 46:2; TAG 14:0–16:1–16:1	796.11	188.16	59.54	166.71	71.52
105	TAG 46:3; TAG 14:0–16:0–16:3	196.66	55.17	20.22	45.05	21.94
106	TAG 46:4; TAG 14:0–14:0–18:4	146.48	42.47	21.75	26.79	16.19
107	TAG 46:5; TAG 12:0–14:0–20:5	38.35	15.58	5.35	9.55	5.17
108	TAG 47:0; TAG 14:0–16:0–17:0	699.02	274.51	68.64	82.03	26.54
109	TAG 47:1; TAG 14:0–16:0–17:1	1540.81	441.21	124.84	306.27	110.83
110	TAG 47:2; TAG 14:0–16:1–17:1	645.66	213.60	69.64	200.66	107.67
111	TAG 47:3; TAG 14:0–16:1–17:2	185.15	63.33	28.77	62.32	48.94
112	TAG 48:0; TAG 16:0–16:0–16:0	2360.71	1349.53	303.78	244.57	66.12
113	TAG 48:1; TAG 14:0–16:0–18:1	11,382.75	2785.59	494.86	1421.02	362.58
114	TAG 48:2; TAG 14:0–16:1–18:1	4745.03	1141.24	257.93	1056.63	354.85
115	TAG 48:3; TAG 14:0–16:1–18:2	1277.27	375.59	120.75	329.42	162.50
116	TAG 48:4; TAG 14:0–16:0–18:4	899.02	311.34	140.68	213.37	101.40
117	TAG 48:5; TAG 14:0–14:0–20:	766.67	218.83	87.21	157.30	81.95
118	TAG 48:6; TAG 12:0–14:0–22:6	117.25	44.23	23.35	36.59	23.90
119	TAG 49:0; TAG 16:0–16:0–17:0	589.05	275.28	55.16	80.23	21.36
120	TAG 49:1; TAG 15:0–16:0–18:1	4237.26	1030.17	202.59	717.88	218.71
121	TAG 49:3; TAG 16:0–16:1–17:2	934.22	320.91	106.11	354.32	246.93
122	TAG 49:5; TAG 14:0–15:0–20:5	422.49	164.69	69.06	135.40	86.89
123	TAG 49:6; TAG 13:0–14:0–22:6	129.64	50.46	31.32	46.43	39.70
124	TAG 50:0; TAG 16:0–16:0–18:0	1232.27	602.91	117.00	142.30	32.46
125	TAG 50:1; TAG 16:0–16:0–18:1	16,831.89	4421.03	701.19	2404.55	622.86
126	TAG 50:2; TAG 16:0–16:1–18:1	13,668.03	3233.82	513.71	3305.07	1136.30
127	TAG 50:3; TAG 16:0–16:1–18:2	4249.84	1245.11	317.65	1371.66	613.79
128	TAG 50:4; TAG 16:0–16:0–18:4	3483.53	1296.83	479.61	893.20	437.83
129	TAG 50:5; TAG 14:0–16:0–20:5	4995.87	1821.60	693.31	1157.60	617.92
130	TAG 50:6; TAG 14:0–14:0–22:6	2948.29	1089.61	545.24	773.05	429.69
131	TAG 50:7; TAG 14:1–16:1–20:5	284.88	109.26	55.64	102.36	85.94
132	TAG 50:8; TAG 14:0–16:3–20:5	61.26	30.70	17.50	22.24	19.88
133	TAG 51:0; TAG 16:0–17:0–18:0	266.67	106.15	22.69	37.34	8.97
134	TAG 51:1; TAG 16:0–17:0–18:1	3277.37	758.78	109.50	490.42	138.98
135	TAG 51:2; TAG 16:0–17:1–18:1	4624.51	1167.22	212.05	1362.95	710.33
136	TAG 51:3; TAG 16:0–18:1–17:2	2132.11	727.84	189.30	870.67	645.99
137	TAG 51:4; TAG 15:0–17:2–19:2	1298.42	509.95	179.67	507.96	362.90
138	TAG 51:5; TAG 15:0–16:0–20:5	1920.43	808.25	323.31	613.60	401.60
139	TAG 51:6; TAG 14:0–15:0–22:6	1716.64	697.80	369.91	594.44	435.23
140	TAG 51:7; TAG 14:0–15:1–22:6	289.07	125.98	73.70	135.69	140.98
141	TAG 52:0; TAG 16:0–18:0–18:0	359.61	136.53	33.57	45.60	11.07
142	TAG 52:1; TAG 16:0–18:0–18:1	7521.60	1610.37	221.37	965.94	225.14
143	TAG 52:10; TAG 14:0–16:4–22:	79.38	42.29	28.41	36.82	38.37
144	TAG 52:2; TAG 16:0–18:1–18:1	14,528.15	3581.68	492.08	3886.73	1487.54
145	TAG 52:3; TAG 16:0–18:1–18:2	6715.38	1893.02	368.07	2336.10	1295.27
146	TAG 52:5; TAG 16:0–16:0–20:5	12,624.93	5019.98	1572.51	3005.89	2075.81
147	TAG 52:6; TAG 14:0–16:0–22:6	20,775.69	8667.66	4465.53	5173.09	2905.92
148	TAG 52:7; TAG 14:0–16:1–22:6	5268.18	2262.78	1127.22	2161.61	1479.09
149	TAG 52:8; TAG 14:0–16:2–22:6	790.22	318.02	176.69	317.53	282.02
150	TAG 52:9; TAG 14:0–18:4–20:5	314.17	147.07	85.42	118.05	120.07
151	TAG 53:1; TAG 17:0–18:0–18:1	1082.49	231.67	36.50	156.72	47.08
152	TAG 53:2; TAG 18:0–17:1–18:1	2097.92	502.91	71.64	535.59	250.17
153	TAG 53:3; TAG 17:1–18:1–18:1	1789.09	570.71	99.01	712.15	605.64
154	TAG 53:5; TAG 16:0–17:0–20:5	2129.93	921.36	315.77	708.85	440.68
155	TAG 53:6; TAG 15:0–16:0–22:6	7756.86	3376.25	1459.65	2259.20	1711.91
156	TAG 53:7; TAG 14:0–17:1–22:6	2959.02	1394.96	830.27	1482.14	1400.46
157	TAG 53:9; TAG 15:0–18:4–20:5	133.67	68.36	44.99	72.04	96.88
158	TAG 54:0; TAG 16:0–18:0–20:0	134.22	47.68	9.95	14.77	4.95
159	TAG 54:1; TAG 18:0–18:0–18:1	1860.35	368.00	61.44	201.70	49.50
160	TAG 54:10; TAG 14:0–18:4–22:6	1354.79	722.54	460.66	578.86	644.29
161	TAG 54:11; TAG 12:0–20:5–22:6	107.87	62.86	44.94	69.50	92.07
162	TAG 54:2; TAG 18:0–18:1–18:1	4348.16	972.05	134.50	996.12	364.78
163	TAG 54:3; TAG 18:1–18:1–18:1	4415.34	1183.47	251.01	1778.01	1051.09
164	TAG 54:4; TAG 18:1–18:1–18:2	4817.22	1802.51	483.94	1553.44	933.86
165	TAG 54:5; TAG 16:0–18:1–20:4	10,617.33	4124.18	1116.78	2678.88	1503.99
166	TAG 54:6; TAG 16:0–16:0–22:6	43,768.75	19,221.93	9247.44	11,928.87	6974.94
167	TAG 54:7; TAG 16:0–16:1–22:6	26,437.66	12,513.05	6802.23	11,540.54	8925.58
168	TAG 54:8; TAG 16:1–16:1–22:6	5369.36	2468.51	1318.56	2998.23	2620.45
169	TAG 54:9; TAG 16:0–18:4–20:5	2226.27	1074.58	596.86	947.74	933.05
170	TAG 55:10; TAG 15:0–18:4–22:6	442.13	245.52	161.68	240.29	327.21
171	TAG 55:2; TAG 19:0–18:1–18:1	596.37	147.34	23.80	134.01	55.29
172	TAG 55:3; TAG 17:1–18:1–20:1	550.48	142.05	42.07	154.17	104.63
173	TAG 55:3; TAG 18:1–18:1–19:1	550.48	142.05	42.07	154.17	104.63
174	TAG 55:9; TAG 16:1–17:2–22:6	750.36	457.78	292.78	569.79	748.54
175	TAG 56:0; TAG 16:0–18:0–22:0	73.62	26.11	5.72	8.15	3.22
176	TAG 56:1; TAG 16:0–22:0–18:1	813.79	179.75	27.99	72.28	18.02
177	TAG 56:10; TAG 16:0–20:5–20:5	7474.70	4374.40	2651.81	3682.49	4208.50
178	TAG 56:11; TAG 14:0–20:5–22:6	3760.87	2100.03	1467.95	2042.91	2572.65
179	TAG 56:12; TAG 14:1–20:5–22:6	168.04	104.55	79.00	119.66	212.57
180	TAG 56:13; TAG 18:4–18:4–20:5	38.44	32.73	20.69	28.38	51.49
181	TAG 56:14; TAG 16:4–20:5–20:5	16.15	13.29	10.86	17.08	23.92
182	TAG 56:2; TAG 16:0–18:1–22:1	1414.86	295.72	48.61	218.14	68.55
183	TAG 56:3; TAG 18:1–18:1–20:1	883.75	245.01	44.24	280.18	145.27
184	TAG 56:6; TAG 16:0–18:0–22:6	23,151.74	8969.90	3069.05	6220.28	3841.85
185	TAG 56:7; TAG 16:0–18:1–22:6	39,424.14	18,549.03	9210.04	17,954.94	14,218.79
186	TAG 56:8; TAG 16:1–18:1–22:6	13,568.57	6887.47	3582.07	8914.80	9537.98
187	TAG 57:12; TAG 13:0–22:6–22:6	168.23	108.44	91.90	125.12	269.09
188	TAG 57:7; TAG 17:0–18:1–22:6	5114.37	2107.36	836.21	2249.61	1799.39
189	TAG 57:8; TAG 17:1–18:1–22:6	3652.72	1716.98	902.90	2740.44	3447.92
190	TAG 58:1; TAG 16:0–24:0–18:1	338.55	74.96	12.09	36.58	10.28
191	TAG 58:13; TAG 16:2–20:5–22:6	355.68	229.28	174.78	262.57	556.77
192	TAG 58:14; TAG 18:4–20:5–20:5	125.61	99.31	75.83	95.27	225.71
193	TAG 58:15; TAG 16:4–20:5–22:6	33.78	24.69	21.50	29.38	69.29
194	TAG 58:2; TAG 16:0–18:1–24:1	856.05	181.40	25.10	119.13	34.12
195	TAG 58:3; TAG 18:1–18:1–22:1	404.50	127.01	27.69	101.60	45.62
196	TAG 58:6; TAG 18:0–18:0–22:6	4769.40	1641.79	392.38	803.00	255.79
197	TAG 58:8; TAG 18:1–18:1–22:6	10,173.23	5079.44	2388.11	7777.92	9902.09
198	TAG 59:13; TAG 17:2–20:5–22:6	275.89	196.08	176.57	232.30	591.30
199	TAG 59:8; TAG 18:1–19:1–22:6	756.99	420.83	141.48	524.42	609.72
200	TAG 60:1; TAG 18:0–24:0–18:1	64.53	15.79	4.00	10.49	3.35
201	TAG 60:12; TAG 16:0–22:6–22:6	32,368.12	20,252.57	15,274.98	16,825.77	26,280.06
202	TAG 60:14; TAG 18:3–20:5–22:6	720.74	506.00	403.96	558.30	1380.37
203	TAG 60:15; TAG 18:4–20:5–22:6	440.17	349.36	275.19	339.00	974.30
204	TAG 60:16; TAG 16:4–22:6–22:6	30.95	27.57	26.66	33.00	107.17
205	TAG 60:2; TAG 16:0–18:1–26:1	210.54	52.11	9.86	42.60	14.43
206	TAG 60:3; TAG 18:1–18:1–24:1	161.87	53.45	14.23	55.31	30.67
207	TAG 60:6; TAG 16:0–22:0–22:6	1778.39	557.32	121.70	262.37	110.80
208	TAG 60:8; TAG 18:1–20:1–22:6	2034.68	1015.92	367.64	1177.28	1123.11
209	TAG 61:14; TAG 17:2–22:6–22:6	296.12	225.53	254.44	286.81	795.73
210	TAG 61:15; TAG 20:5–20:5–21:5	27.26	22.33	28.14	16.53	105.81
211	TAG 61:16; TAG 19:5–20:5–22:6	13.75	16.23	10.37	11.45	33.84
212	TAG 62:1; TAG 16:0–22:0–24:1	13.79	4.77	3.77	2.83	1.20
213	TAG 62:13; TAG 18:1–22:6–22:6	9988.21	7303.93	6706.18	9792.43	20,030.62
214	TAG 62:16; TAG 20:5–20:5–22:6	1432.25	1074.75	909.81	1098.48	3579.97
215	TAG 62:6; TAG 16:0–24:0–22:6	918.32	250.03	71.31	149.45	65.83
216	TAG 62:8; TAG 18:1–22:1–22:6	963.28	405.18	118.21	376.57	314.10
217	TAG 63:13; TAG 19:1–22:6–22:6	215.93	177.08	142.80	199.15	373.42
218	TAG 63:16; TAG 20:5–21:5–22:6	60.25	58.39	56.25	56.81	194.69
219	TAG 63:8; TAG 17:1–24:1–22:6	182.90	74.56	25.59	83.02	54.52
220	TAG 64:13; TAG 20:1–22:6–22:6	688.32	546.05	463.50	656.28	1070.28
221	TAG 64:14; TAG 20:2–22:6–22:6	271.53	154.26	177.03	203.52	366.76
222	TAG 64:16; TAG 20:4–22:6–22:6	1275.00	1169.85	1275.64	1359.68	5210.51
223	TAG 64:17; TAG 20:5–22:6–22:6	2502.17	2126.66	2061.56	2254.17	8625.88
224	TAG 64:6; TAG 18:0–24:0–22:6	159.58	52.76	16.15	31.74	17.58
225	TAG 64:9; TAG 18:1–24:2–22:6	198.84	105.08	40.08	98.33	77.03
226	TAG 65:12; TAG 21:0–22:6–22:6	73.03	39.23	29.22	23.18	28.92
227	TAG 65:17; TAG 21:5–22:6–22:6	62.58	57.91	66.92	61.74	246.85
228	TAG 66:12; TAG 24:1–20:5–22:6	290.05	211.80	141.48	217.40	287.21
229	TAG 66:18; TAG 22:6–22:6–22:6	1394.71	1374.35	1716.92	1866.91	9604.45
230	TAG O-47:1; TAG O-17:0–14:0–16:1	64.26	25.58	8.49	16.30	6.54
231	TAG O-49:3; TAG O-15:0–16:0–18:3	65.96	23.33	8.00	22.19	12.64
232	TAG O-49:4; TAG O-15:0–16:1–18:3	46.79	19.10	8.46	13.86	8.12
233	TAG O-51:0; TAG O-19:0–16:0–16:0	36.39	20.33	7.44	7.64	3.69
234	TAG O-51:6; TAG O-15:0–14:0–22:6	89.92	46.29	25.42	43.59	31.91
235	TAG O-53:2; TAG O-17:0–18:1–18:1	221.70	87.80	29.96	81.23	57.17
236	TAG O-53:8; TAG O-15:0–16:2–22:6	64.22	23.73	14.06	25.65	25.34
237	TAG O-54:3; TAG O-18:1–18:1–18:1	97.14	35.22	9.11	49.46	35.56
238	TAG O-55:5; TAG O-17:0–16:0–22:5	502.38	263.82	106.03	208.61	150.81
239	TAG O-57:7; TAG O-17:0–18:1–22:6	1150.46	712.15	244.95	694.66	552.02
240	TAG O-61:12; TAG O-17:0–22:6–22:6	1529.10	1071.69	708.75	987.05	1475.27
241	MAG 14:1	0.78	3.56	3.49	1.98	2.49
242	MAG 16:0	127.53	997.54	199.96	179.46	532.42
243	MAG 18:0	277.94	545.64	283.62	226.69	171.56
244	MAG 20:4	0.00	4228.83	646.32	1196.14	763.06
245	MAG 20:5	11.28	11,143.98	1604.80	3154.08	2149.15
246	MAG 22:5	0.00	10,449.60	3095.61	1905.45	1082.52
247	MAG 22:6	4.88	101,998.89	40,898.48	16,129.83	10,331.57
TAG		478,220.42	201,130.27	97,880.59	176,850.18	185,560.95
DAG		15,174.52	614,859.76	571,876.83	336,213.70	720,765.00
MAG		422.41	129,368.04	46,732.27	22,793.64	15,032.77
